# Effect of Underlying Cardiometabolic Diseases on the Association Between Sedentary Time and All‐Cause Mortality in a Large Japanese Population: A Cohort Analysis Based on the J‐MICC Study

**DOI:** 10.1161/JAHA.120.018293

**Published:** 2021-06-14

**Authors:** Teruhide Koyama, Etsuko Ozaki, Nagato Kuriyama, Satomi Tomida, Tamami Yoshida, Ritei Uehara, Keitaro Tanaka, Megumi Hara, Asahi Hishida, Rieko Okada, Yoko Kubo, Isao Oze, Yuriko N. Koyanagi, Haruo Mikami, Yohko Nakamura, Ippei Shimoshikiryo, Toshiro Takezaki, Sadao Suzuki, Takahiro Otani, Kiyonori Kuriki, Naoyuki Takashima, Aya Kadota, Kokichi Arisawa, Sakurako Katsuura‐Kamano, Hiroaki Ikezaki, Masayuki Murata, Kenji Takeuchi, Kenji Wakai, the Japan Multi‐Institutional Collaborative Cohort (J‐MICC) Study Group, Hiroki Nagase, Hiroto Narimatsu, Keitaro Matsuo, Yoshikuni Kita, Katsuyuki Miura

**Affiliations:** ^1^ Department of Epidemiology for Community Health and Medicine Kyoto Prefectural University of Medicine Kyoto Japan; ^2^ Department of Endocrine and Breast Surgery Kyoto Prefectural University of Medicine Kyoto Japan; ^3^ Shizuoka Graduate University of Public Health Shizuoka Japan; ^4^ Department of Preventive Medicine Faculty of Medicine Saga University Saga Japan; ^5^ Department of Preventive Medicine Nagoya University Graduate School of Medicine Aichi Japan; ^6^ Division of Cancer Epidemiology and Prevention Aichi Cancer Center Research Institute Aichi Japan; ^7^ Division of Cancer Information and Control Aichi Cancer Center Research Institute Aichi Japan; ^8^ Cancer Prevention Center Chiba Cancer Center Research Institute Chiba Japan; ^9^ Department of International Island and Community Medicine Kagoshima University Graduate School of Medical and Dental Sciences Kagoshima Japan; ^10^ Department of Public Health Nagoya City University Graduate School of Medical Sciences Aichi Japan; ^11^ Laboratory of Public Health School of Food and Nutritional Sciences University of Shizuoka Japan; ^12^ Department of Public Health Faculty of Medicine Kindai University Osaka Japan; ^13^ Department of Public Health Shiga University of Medical Science Shiga Japan; ^14^ Department of Preventive Medicine Tokushima University Graduate School of Biomedical Sciences Tokushima Japan; ^15^ Department of General Internal Medicine Kyushu University Hospital Fukuoka Japan; ^16^ Department of Comprehensive General Internal Medicine Faculty of Medical Sciences Kyushu University Fukuoka Japan

**Keywords:** all‐cause mortality, diabetes mellitus, dyslipidemia, hypertension, sedentary time, Epidemiology, Cardiovascular Disease

## Abstract

**Background:**

This study aimed to determine the association between sedentary time and mortality with regard to leisure‐time physical activity with or without cardiometabolic diseases such as hypertension, dyslipidemia, and diabetes mellitus.

**Methods and Results:**

Using data from the J‐MICC (Japan Multi‐Institutional Collaborative Cohort) Study, 64 456 participants (29 022 men, 35 434 women) were analyzed. Hazard ratios (HRs) and 95% CIs were used to characterize the relative risk of all‐cause mortality to evaluate its association with sedentary time (categorical variables: <5, 5 to <7, 7 to <9, ≥9 h/d and 2‐hour increments in exposure) according to the self‐reported hypertension, dyslipidemia, and diabetes mellitus using a Cox proportional hazards model. A total of 2257 participants died during 7.7 years of follow‐up. The corresponding HRs for each 2‐hour increment in sedentary time among participants with all factors, no factors, hypertension, dyslipidemia, and diabetes mellitus were 1.153 (95% CI, 1.114–1.194), 1.125 (95% CI, 1.074–1.179), 1.202 (95% CI, 1.129–1.279), 1.176 (95% CI, 1.087–1.273), and 1.272 (95% CI, 1.159–1.396), respectively. Furthermore, when analyzed according to the combined different factors (hypertension, dyslipidemia, and diabetes mellitus), HRs increased with each additional factor, and participants reporting all 3 conditions had the highest HR of 1.417 (95% CI, 1.162–1.728) independently of leisure‐time metabolic equivalents.

**Conclusions:**

The association between sedentary time and increased mortality is stronger among patients with hypertension, dyslipidemia, and diabetes mellitus regardless of leisure‐time physical activity in a large Japanese population.

**Nonstandard Abbreviations and Acronyms**
J‐MICC: Japan Multi‐Institutional Collaborative Cohort
LT‐METs: leisure‐time metabolic equivalents

**Clinical Perspective**

**What Is New?**

- The association between sedentary time and increased mortality is stronger among participants with cardiometabolic diseases related to hypertension, dyslipidemia, and diabetes mellitus regardless of leisure‐time metabolic equivalents in a large population‐based cohort study of Japanese adults.

**What Are the Clinical Implications?**

- The association between sedentary time and the number of hypertension/dyslipidemia/diabetes mellitus traits increases the risk of mortality.
- The association between sedentary time and increased mortality persists regardless of leisure‐time physical activity.
- Greater health literacy is needed to interrupt and reduce sedentary time in adults to promote preventive medicine.

Physical inactivity is recognized as a pandemic that requires global attention. Physical inactivity and its harmful effects on health is highly prevalent worldwide. The evidence of the effectiveness of physical activity promotion strategies make this problem a global public health priority.^1^ Physical inactivity is also significant from the perspective of economic burden.^2^ Some systematic reviews and meta‐analyses on physical inactivity reported that sedentary behavior (sitting or reclining posture) is associated with negative health consequences^3^ including increased cardiovascular‐specific, cancer‐specific, and overall mortality.^4^, ^5^, ^6^ Although modest amounts of activity provide substantial benefits for postponing mortality,^7^ it is unclear whether leisure‐time physical activity offsets the health risks of sedentary behavior.

Recently, we reported the association of sedentary time with cardiometabolic diseases such as hypertension, dyslipidemia, and diabetes mellitus in a large Japanese population.^8^ However, there are no studies on the association between sedentary time and mortality in patients with cardiometabolic diseases such as hypertension, dyslipidemia, and diabetes mellitus. Previous studies have reported that sedentary time affects cardiovascular diseases^9^ and is associated with mortality.^4^, ^10^, ^11^ In sum, the hypothesis holds that longer sedentary times tend to increase the risk of death in patients with cardiometabolic diseases. On the other hand, although the critical mechanism is unknown, several studies have shown that sedentary behavior–related health disorders are independent of physical activity.^12^, ^13^ Actually, our previous study showed sedentary time was associated with cardiometabolic diseases independent of leisure‐time physical activity.^8^ This study therefore aimed to determine the association between sedentary time and mortality with regard to leisure‐time physical activity with or without underlying diseases such as hypertension, dyslipidemia, and diabetes mellitus in a large Japanese population.

## Methods

Requests to access the data set should be consulted with the J‐MICC (Japan Multi‐Institutional Collaborative Cohort) Study central office via the corresponding author of this article.

### Study Participants

In the present study, we evaluated participant data collected during the J‐MICC Study.^14^ The cohort study collected and analyzed genetic and clinical data from the general Japanese population to detect and confirm gene–environment interactions related to lifestyle‐associated diseases. The study participants were aged 35 to 69 years and were enrolled upon responding to study announcements, attending health checkup examinations commissioned by their local governments, visiting local health checkup centers, or visiting a cancer hospital from February 2004 through March 2014.

When there were no data on participants' daily activities including sitting time, the research sites were excluded. Then, we excluded participants who lacked the following data in the self‐administered questionnaire: history of hypertension, dyslipidemia, or diabetes mellitus; smoking and drinking status; physical activity including sitting time; the medical history of ischemic heart disease and stroke; drug treatment for hypertension, dyslipidemia, or diabetes mellitus; and those followed up for <1 year, which was too short of a period to assess the daily impact of reduced physical activity caused by the disease.

Changes in residence status, including vital status, were confirmed from 2004 using the residential registry. Mortality data were obtained from the Ministry of Health, Labor, and Welfare of Japan, and the underlying causes of death were coded according to the *International Classification of Diseases*, *Tenth Revision (ICD‐10*). Registration of death was required by the Family Registration Law and was believed to be complete, except for subjects who died after moving from their original study area. The participants were followed up through December 2016 in 10 study areas or December 2017 in 4 areas, except for parts of 1 area (December 2012 or December 2013). Selection bias was avoided because sufficient follow‐up was conducted by the research sites.

All study participants provided written informed consent. The study protocol was approved by the ethics committees at the Nagoya University Graduate School of Medicine (IRB No. 2010‐939) and other institutions participating in the J‐MICC Study. This study was conducted according to the principles enshrined in the World Medical Association Declaration of Helsinki.

### Self‐Administered Questionnaire Data

In this study, we evaluated data on clinical and lifestyle variables (smoking and drinking status, and physical activity including sitting time) obtained through self‐administered questionnaires. Physical activity was determined using a format similar to that of the International Physical Activity Questionnaire.^15^ Leisure‐time physical activity was measured in terms of leisure‐time metabolic equivalents (LT‐METs), as previously reported.^16^, ^17^ In brief, metabolic equivalent hours per day of leisure‐time activity were estimated by multiplying the reported daily time spent in each activity by its corresponding metabolic equivalent intensity. We divided the LT‐METs groups into quartiles (Q1–Q4). The duration of sitting time was asked with 8 possible responses: none, <1, 1 to <3, 3 to <5, 5 to <7, 7 to <9, 9 to <11, and ≥11 h/d. Sitting time was then categorized on the basis of the quartile value as mentioned in a previous study^8^: <5, 5 to <7, 7 to <9, or ≥9 h/d. Medical history and medication history were assessed using self‐administered questionnaires. Hypertension, dyslipidemia (specifically hyperlipidemia), and diabetes mellitus were defined by the presence or absence of medical history and/or current use of medication.

### Statistical Analysis

Continuous variables were expressed as means and SDs, and categorical variables were expressed as frequencies and percentages. Intergroup comparisons were performed using 1‐way ANOVA for continuous variables and χ^2^ tests for categorical variables. Person‐years of follow‐up were calculated from the date of filling out the baseline questionnaire to the time of death, moving out of the area, or the end of the follow‐up, whichever came first. Hazard ratios (HRs) and 95% CIs were used to characterize the relative risk of all‐cause mortality to evaluate its association with sedentary time (<5 hours was used as a reference; categorical variables: <5, 5 to <7, 7 to <9, ≥9 and 2‐hour increments in exposure) according to the self‐reported hypertension, dyslipidemia, and diabetes mellitus using a Cox proportional hazards model. The model was adjusted for age (operationalized as a continuous variable), sex, research area, LT‐METs (Q1–Q4), drinking and smoking status (never, former, and current), and history of ischemic heart disease and stroke, and history of medication for hypertension, dyslipidemia, and diabetes mellitus. The incidence of mortality was calculated using the Kaplan‐Meier method. Tests for linear trends (eg, *P* trend tests) were conducted by including 4 groups according to LT‐METs (Q1–Q4) as ordinal variables. Using G*Power (http://www.gpower.hhu.de), we verified that the sample size was sufficient. A post hoc power calculation showed the sample size in this study achieved a power of 0.80 at 0.05 α level for a 2‐sided test. All statistical analyses were performed using SPSS software version 25 (IBM Japan, Tokyo, Japan) and JMP 13 software (SAS Institute, Cary, NC), and *P*<0.005 was considered to indicate statistical significance.

## Results

**Figure 1:**
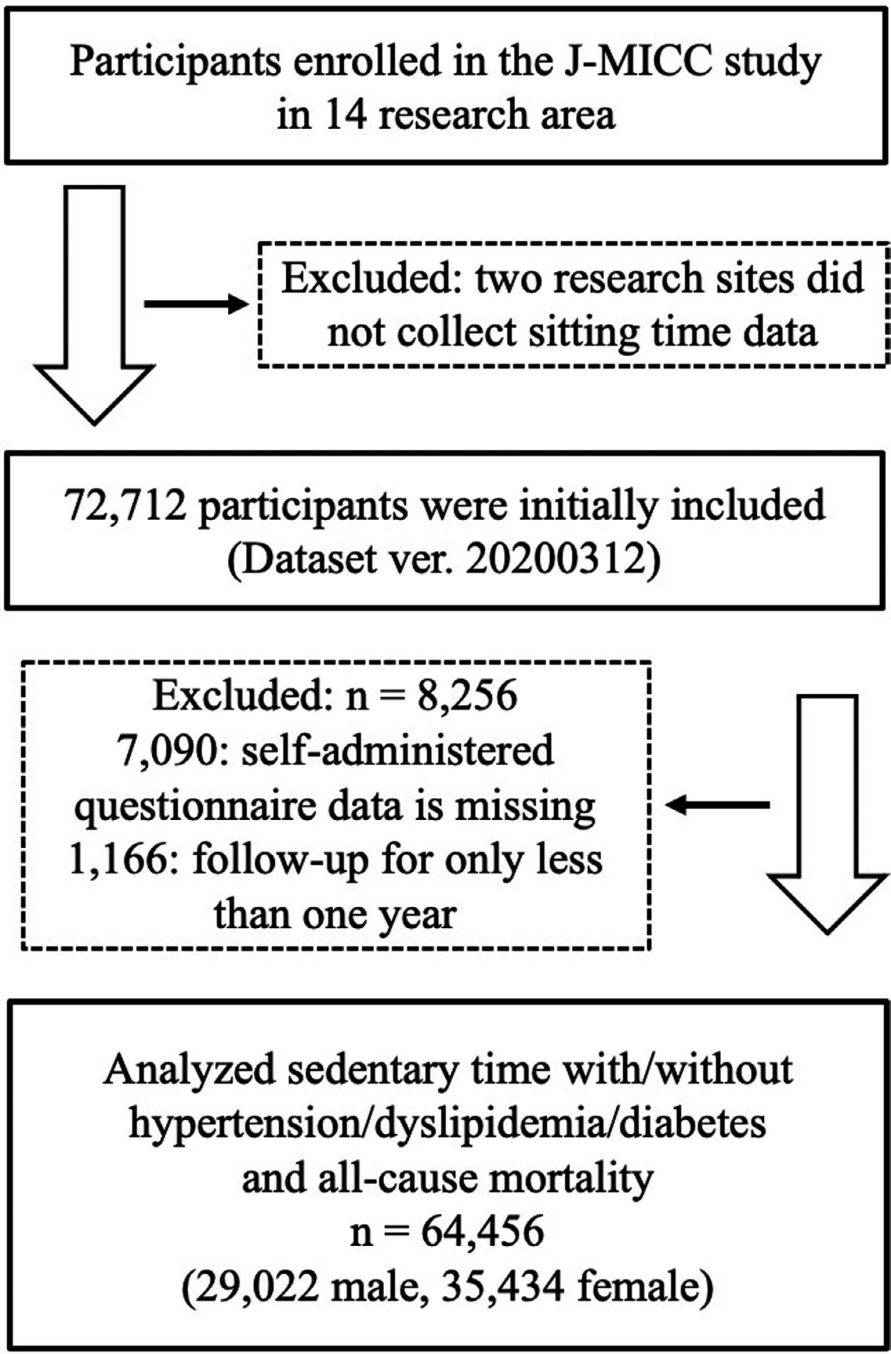
Flowchart of the study participants. J‐MICC indicates Japan Multi‐Institutional Collaborative Cohort.

Figure 1 shows the study participant flowchart. Of the 14 research sites, 2 did not collect data on participants' daily activities including sitting time. Excluding the participants from these 2 research sites, 72 712 participants were initially included in the current study (data set version 20200312). We verified the reproducibility of the analysis results and confirmed that the results were correct.

Participants' characteristics, including drinking and smoking status, medical history of hypertension/dyslipidemia/diabetes mellitus, and distribution of age and sex according to sedentary time are presented in Table 1. Of 64 456 participants included in the current analysis, 24 304 (37.7%), 14 596 (22.6%), 10 481 (16.3%), and 15 075 (23.4%) spent <5, 5 to <7, 7 to <9, and ≥9 hours per day being sedentary, respectively. A total of 2257 participants died during the 7.7 years (mean) of follow‐up. Mortality increased with longer sedentary time, and the mortality rate (cases per 1000 person‐years) increased from 3.93 in the <5‐hours group to 6.26 in the ≥9‐hours group.

**Table 1:** Characteristics of Participants According to Sedentary Time

Sedentary Time	<5 h	5 to <7 h	7 to <9 h	≥9 h
	n=24 304	n=14 596	n=10 481	n=15 075
Age, y	54.7±9.45	55.4±9.43	54.8±9.55	53.4±9.64
Sex, men	9910 (40.8)	5719 (39.2)	4678 (44.6)	8715 (57.8)
No. of deaths	789 (3.2)	449 (3.1)	366 (3.5)	653 (4.3)
Person‐years	200 658	114 237	79 389	104 386
Mortality rate (per 1000 person‐years)	3.93	3.93	4.61	6.26
Drinking status				
Current	13 261 (54.6)	8003 (54.8)	6022 (57.5)	9318 (61.8)
Former	492 (2.0)	352 (2.4)	249 (2.4)	465 (3.1)
Never	10 551 (43.4)	6241 (42.8)	4210 (40.2)	5292 (35.1)
Smoking status				
Current	4226 (17.4)	2157 (14.8)	1606 (15.3)	2885 (19.1)
Former	4990 (20.5)	3115 (21.3)	2537 (24.2)	4394 (29.1)
Never	15 088 (62.1)	9324 (63.9)	6338 (60.5)	7796 (51.7)
Hypertension	4697 (19.3)	3007 (20.6)	2178 (20.8)	3078 (20.4)
Dyslipidemia	4010 (16.5)	2928 (20.1)	2123 (20.3)	3124 (20.7)
Diabetes mellitus	1380 (5.7)	845 (5.8)	659 (6.3)	1005 (6.7)
Stroke	366 (1.5)	227 (1.6)	159 (1.5)	258 (1.7)
IHD	590 (2.4)	366 (2.5)	316 (3.0)	471 (3.1)
LT‐METs, h/d	2.21±3.54	2.32±3.23	2.06±2.89	1.71±2.52

Data are presented as mean±SD or number (percentage). IHD indicates ischemic heart disease; and LT‐METs, leisure‐time metabolic equivalents.

**Figure 2:**
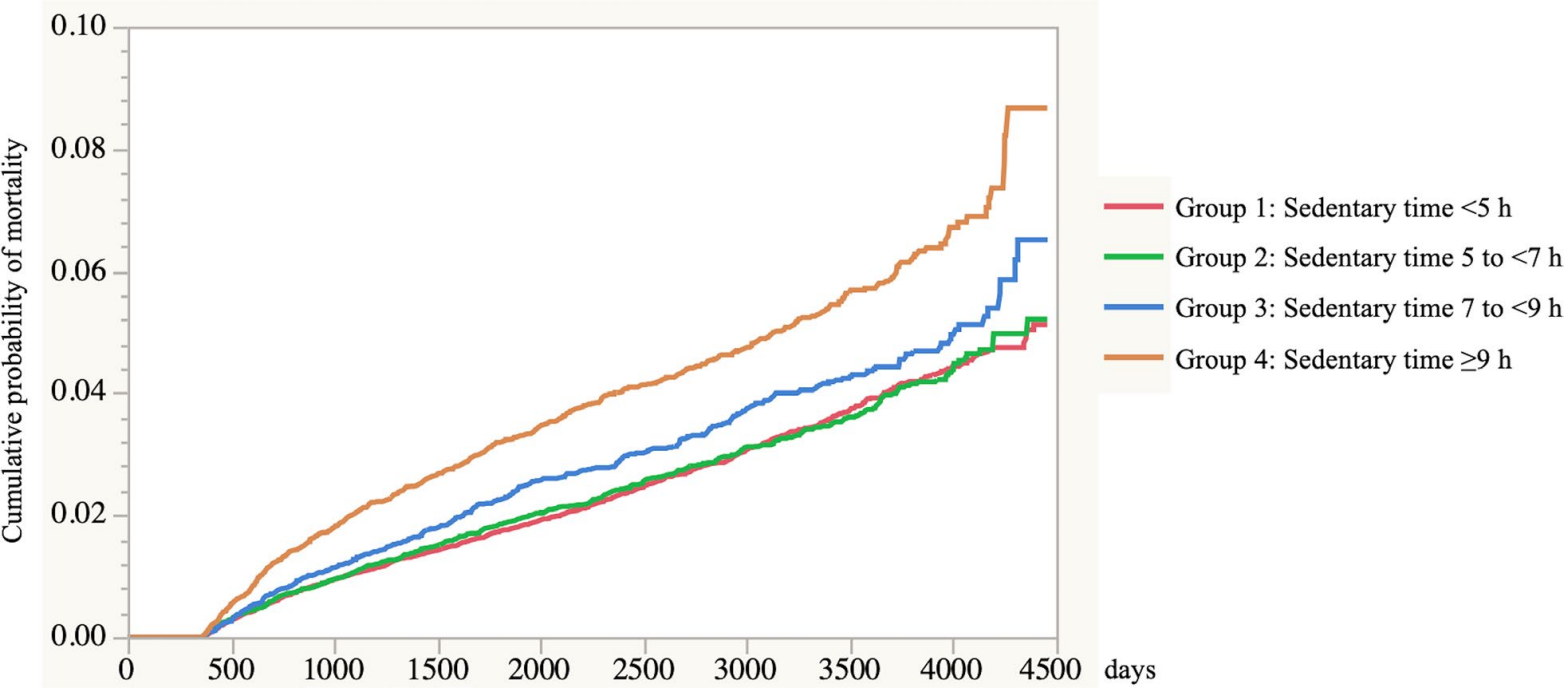
Kaplan‐Meier cumulative mortality classified by sedentary time (Group 1: <5 hours; Group 2: 5 to <7 hours; Group 3: 7 to <9 hours; and Group 4: ≥9 hours). *P*<0.001 using the log‐rank test.

Results from the multivariate model (ie, adjusted HR [95% CI]) of all‐cause mortality according to medical history of hypertension, dyslipidemia, and diabetes mellitus are shown in Table 2. Compared with participants who spent <5 hours of sedentary time, those who had longer sedentary time tended to have significantly higher HRs, especially in the ≥9‐hours group. The corresponding HR for each 2‐hour increment in sedentary time was 1.153 (95% CI, 1.114–1.194) among all participants. The corresponding HRs for each 2‐hour increment in sedentary time among participants with no factors, hypertension, dyslipidemia, and diabetes mellitus were 1.125 (95% CI, 1.074–1.179), 1.202 (95% CI, 1.129–1.279), 1.176 (95% CI, 1.087–1.273), and 1.272 (95% CI, 1.159–1.396), respectively. Furthermore, when analyzed according to the combined different factors (hypertension, dyslipidemia, and diabetes mellitus), HRs increased with each additional factor, and participants reporting all 3 conditions had the highest HR of 1.417 (95% CI, 1.162–1.728). The Kaplan‐Meier cumulative mortality data stratified by group are shown in Figure 2. The risk of mortality significantly increased among the group with a longer sedentary time (*P*<0.001 using the log‐rank test).

**Table 2:** Associations Between Death and Sedentary Time According to Self‐Reported Cardiometabolic Disease

	No.	No. of Deaths	Sedentary Time							2‐h Increments in Sedentary Time	
			<5 h	5 to <7 h		7 to <9 h		≥9 h			
			Reference	HR	95% CI	HR	95% CI	HR	95% CI	HR	95% CI
All	64 456	2257	1.000	1.031	0.918–1.158	1.205	1.064–1.364	1.540	1.386–1.712	1.153	1.114–1.194
Hypertension	12 960	702	1.000	1.075	0.868–1.329	1.328	1.063–1.659	1.729	1.430–2.090	1.202	1.129–1.279
Dyslipidemia	12 185	454	1.000	1.133	0.871–1.453	1.405	1.069–1.847	1.608	1.259–2.055	1.176	1.087–1.273
Diabetes mellitus	3889	306	1.000	1.566	1.130–2.170	1.728	1.225–2.438	2.142	1.594–2.877	1.272	1.159–1.396
No. of hypertension/dyslipidemia/diabetes mellitus											
None	42 911	1231	1.000	1.017	0.871–1.188	1.129	0.953–1.339	1.447	1.254–1.668	1.125	1.074–1.179
1	15 067	666	1.000	0.896	0.719–1.117	1.214	0.968–1.522	1.619	1.336–1.962	1.181	1.108–1.259
2	5467	284	1.000	1.311	0.943–1.823	1.386	0.967–1.987	1.741	1.282–2.366	1.192	1.081–1.316
3	1011	76	1.000	2.269	1.112–4.630	2.684	1.324–5.437	3.175	1.624–6.210	1.417	1.162–1.728

Adjusted for age, sex, research area, leisure‐time‐metabolic equivalents, drinking and smoking status, ischemic heart disease, stroke, and history of medication for hypertension, dyslipidemia, and diabetes mellitus. HR indicates hazard ratio.

We also reported results of HRs for each 2‐hour increment in sedentary time according to the LT‐METs quartile. Table S1 shows the characteristics of participants in each LT‐METs quartile. As the LT‐METs quartile increased, the mean age and proportion of participants reporting hypertension/dyslipidemia/diabetes mellitus increased; however, mortality rate (cases per 1000 person‐years) decreased with higher LT‐METs from 5.14 in the Q1 group to 4.19 in the Q4 group. In the all‐ and no‐factors groups, participants with higher LT‐METs tended to have significantly lower HRs; particularly in the fourth quartile of LT‐METs (Q4), the HR for the no‐factors group was not significant (HR, 1.091; 95% CI, 0.982–1.211) (Table 3). However, most HRs of sedentary time and mortality remained statistically significant, regardless of the LT‐METs group.

**Table 3:** Associations Between Death and 2‐Hour Increments in Sedentary Time According to LT‐METs Quartile

LT‐METs	Q1		Q2		Q3		Q4		*P* Value for Trend
	HR	95% CI	HR	95% CI	HR	95% CI	HR	95% CI	
All	1.169	1.100–1.242	1.143	1.067–1.226	1.165	1.082–1.254	1.126	1.042–1.217	<0.001
Hypertension	1.257	1.123–1.406	1.187	1.048–1.344	1.162	1.021–1.322	1.177	1.022–1.356	<0.001
Dyslipidemia	1.090	0.945–1.257	1.230	1.045–1.448	1.212	1.022–1.438	1.206	1.026–1.417	<0.001
Diabetes mellitus	1.352	1.151–1.587	1.205	0.992–1.464	1.243	1.033–1.495	1.229	0.965–1.567	<0.001
No. of hypertension/dyslipidemia/diabetes mellitus									
None	1.147	1.058–1.243	1.111	1.012–1.220	1.147	1.035–1.270	1.091	0.982–1.211	<0.001
1	1.197	1.067–1.344	1.165	1.024–1.325	1.212	1.062–1.383	1.157	1.001–1.337	<0.001
2	1.192	0.995–1.428	1.364	1.114–1.671	1.072	0.869–1.323	1.149	0.931–1.418	<0.001
3	1.506	1.066–2.126	1.029	0.664–1.594	1.612	1.071–2.427	2.044	1.138–3.670	0.018

Adjusted for age, sex, research area, drinking and smoking status, ischemic heart disease, stroke and history of medication for hypertension, dyslipidemia, and diabetes mellitus. HR indicates hazard ratio; and LT‐METs, leisure‐time metabolic equivalents.

## Discussion

Considerable evidence suggests that sedentary behavior is associated with increased mortality.^13^, ^18^, ^19^, ^20^ The results of this study confirm the association that each 2‐hour increment in sedentary time was associated with a 15.3% increase in the risk of mortality among all participants and a 41.7% increase in the risk of mortality among participants with hypertension, dyslipidemia, and diabetes mellitus. Thus, our results suggest that the well‐established association between sedentary time and increased mortality is stronger among patients with a medical history of hypertension, dyslipidemia, and diabetes mellitus. In general, common correlates of sedentary behavior are known to differ by ethnicity.^21^ Few studies have examined the association between sedentary time and mortality in Japan. The only 2 studies that showed an association between sedentary time and mortality involved limited situations: television viewing time and mortality from stroke and coronary artery disease,^22^ and occupational sitting time and all‐cause mortality.^23^ To the best of our knowledge, this is the first study to determine that each 2‐hour increment in sedentary time and the number of hypertension/dyslipidemia/diabetes mellitus traits increases the risk of mortality in Japanese adults.

In this study, the population characteristics varied across LT‐METs quartiles, as shown in Table S1. As the LT‐METs quartile increased, the mean age and the proportion of participants with hypertension and/or dyslipidemia and/or diabetes mellitus also increased. Interestingly, the number of deaths decreased with increasing LT‐METs quartile. It was speculated that in the higher‐quartile LT‐METs group, factors such as being elderly and having underlying diseases might have affected health consciousness. In general, the strength of the associations between motivational regulations and physical activity behavior varied across age groups.^24^, ^25^ Health promotion involving physical activity is well established, and researchers have sought to explore the reasons why some people are physically active, whereas others are not.^26^, ^27^, ^28^, ^29^ Older individuals exhibit greater concerns about health outcomes, thus motivating them to perform physical activity.^30^ It is reasonable to expect that older adults would show more concern for physical and psychological health issues when making their physical activity decisions.^31^ Based on these results, the higher quartile LT‐METs group included older subjects and participants with underlying diseases, which may have affected the association of mortality with sedentary time. On the other hand, it is suggested that increasing physical activity during leisure time after the onset of a disease or because of increasing age may be considered a part of secondary or tertiary prevention of the disease, but it is not sufficient for primary prevention. This highlights the necessity of an intervention for the allocation of daily physical activity behaviors among people who do not have a disease and who lack health awareness, for the purpose of promoting preventive medicine. Sedentary time was associated with cardiometabolic diseases such as hypertension, dyslipidemia, and diabetes mellitus among Japanese population.^8^ In sum, reducing sedentary time may be an effective measure to prevent cardiometabolic diseases and death. However, some people cannot change their behavior during working hours. Because sedentary behavior accounts for 55% to 60% of the total duration of daytime activity,^32^ the use of leisure time on weekdays can reduce the duration of sedentary time during the day outside of working hours.

We examined the effect of factors (defined as self‐reported hypertension, dyslipidemia, or diabetes mellitus in this study) on the relationship between sedentary time and risk of death. Sedentary behavior in combination with chronic diseases,^33^ including hypertension^34^ and diabetes mellitus,^20^ increased all‐cause mortality risk. Although the relationship between sedentary time and death among participants with dyslipidemia is often unknown, our results showed that participants with at least 1 factor had a higher risk of mortality than did participants with none. As inferred from previous reports, the association between sedentary time and diabetes mellitus has a stronger risk for mortality than another medical history.^35^ Furthermore, increasing the number of people with hypertension/dyslipidemia/diabetes mellitus increases the risk of mortality (HR), which is as expected. Importantly, a strong association between sedentary time and mortality was observed among participants with hypertension/dyslipidemia/diabetes mellitus independently of LT‐METs grouping; therefore, there is a need to increase awareness among healthy people who lack health literacy to interrupt and reduce their sedentary time.

Despite our novel findings, this study has some limitations. This study used a self‐administered questionnaire at baseline survey to evaluate sedentary time and medical history. Changes in risk factors since the baseline survey could not be taken into account, such as sitting time, exercise during leisure time, drinking, and smoking. Although a questionnaire that evaluates sitting time is controversial, the International Physical Activity Questionnaire is a widely accepted international physical activity surveillance instrument.^36^ Self‐reported measures continue to be the most widely used method for assessing these behaviors. However, evidence from one review suggests that single‐item self‐reported measures generally underestimate sedentary time compared with device‐based measures.^37^ The strength of this study is the inclusion of a large number of participants, which prevented sample bias and implementation of a population‐based cohort design.

## Conclusions

This study showed that the association between sedentary time and increased mortality is stronger among patients with cardiometabolic diseases related to hypertension, dyslipidemia, and diabetes mellitus regardless of LT‐METs in a large Japanese population.

## Appendix

J‐MICC Study Group: Kenji Wakai, Kenji Takeuchi, Haruo Mikami, Hiroki Nagase, Hiroto Narimatsu, Kiyonori Kuriki, Keitaro Matsuo, Sadao Suzuki, Asahi Hishida, Yoshikuni Kita, Katsuyuki Miura, Ritei Uehara, Kokichi Arisawa, Hiroaki Ikezaki, Keitaro Tanaka, and Toshiro Takezaki.

## Sources of Funding

This study was supported by Grants‐in‐Aid for Scientific Research for Priority Areas of Cancer (No. 17015018) and Innovative Areas (No. 221S0001) and by the Japan Society for the Promotion of Science (JSPS) KAKENHI Grant (No. 16H06277 [CoBiA]) from the Japanese Ministry of Education, Culture, Sports, Science and Technology.

## Disclosures

None.

## Supporting information

Table S1
