# Supplementary material for: Effect of Underlying Cardiometabolic Diseases on the Association Between Sedentary Time and All‐Cause Mortality in a Large Japanese Population: A Cohort Analysis Based on the J‐MICC Study
Source: J Am Heart Assoc. 2021 Jun 14;10(13):e018293. doi: 10.1161/JAHA.120.018293 (PMC8403304; doi:10.1161/JAHA.120.018293)
Supplement: Supplementary file 1 — Table S1 [file JAH3-10-e018293-s001.pdf]

SUPPLEMENTAL MATERIAL

Table S1. Characteristics of participants according to LT-METs quartile.

LT-METs		Q1 n = 17021		Q2 n= 16530		Q3 n = 14889		Q4 n = 16016		p-value
Age (year)		52.5	±9.40	53.4	±9.35	55.5	±9.35	57.1	±9.32	<0.001
Sex (men)		7065	41.5%	7177	43.4%	6925	46.5%	7855	49.0%	<0.001
No. of death		669	3.9%	561	3.4%	514	3.5%	513	3.2%	0.003
Person-year		130130		128965		117010		122565		
Mortality rate (/1000 person-years)		5.14		4.35		4.39		4.19		
Drinking status										
	Current	9011	52.9%	9292	56.2%	8617	57.9%	9684	60.5%	
	Former	475	2.8%	387	2.3%	349	2.3%	347	2.2%	<0.001
	Never	7535	44.3%	6851	41.4%	5923	39.8%	5985	37.4%	
Smoking status										
	Current	3759	22.1%	2673	16.2%	2298	15.4%	2144	13.4%	
	Former	3237	19.0%	3738	22.6%	3740	25.1%	4321	27.0%	<0.001
	Never	10025	58.9%	10119	61.2%	8851	59.4%	9551	59.6%	
Hypertension		3003	17.6%	3152	19.1%	3236	21.7%	3569	22.3%	<0.001
Dyslipidemia		2616	15.4%	3049	18.4%	3099	20.8%	3421	21.4%	<0.001
Diabetes		855	5.0%	894	5.4%	1001	6.7%	1139	7.1%	<0.001
Stroke		230	1.4%	242	1.5%	242	1.6%	296	1.8%	0.002
IHD		428	2.5%	408	2.5%	428	2.9%	479	3.0%	0.006

Data are presented as mean (±SD), or number (percentage).

IHD, ischemic heart disease; LT-METs, Leisure-time-metabolic equivalents.
